# Efficacy and Safety of First-Line Immunotherapy in Combination with Chemotherapy for Patients with Extensive-Stage Small Cell Lung Cancer: A Systematic Review and Network Meta-Analysis

**DOI:** 10.1155/2020/2368164

**Published:** 2020-09-29

**Authors:** Bi-Cheng Wang, Bo-Ya Xiao, Peng-Cheng Li, Bo-Hua Kuang, Wang-Bing Chen, Pin-Dong Li, Guo-He Lin, Quentin Liu

**Affiliations:** ^1^Cancer Center, Union Hospital, Tongji Medical College, Huazhong University of Science and Technology, Wuhan 430022, China; ^2^Shanghai Eastern Hepatobiliary Surgery Hospital, Shanghai 200438, China; ^3^Department of Oncology, The Second Affiliated Hospital of Anhui Medical University, Hefei 230601, China; ^4^State Key Laboratory of Oncology in South China, Collaborative Innovation Center for Cancer Medicine, Cancer Center, Sun Yat-sen University, Guangzhou 510060, China

## Abstract

**Background:**

The prognosis of patients with extensive-stage small cell lung cancer (SCLC) is poor. Adding an immune checkpoint inhibitor (ICI) to chemotherapy may exert a synergistic effect and improve survival outcomes. However, for treatment-naive extensive-stage SCLC patients, the efficacy of immunotherapy in combination with cytotoxic chemotherapy remains controversial.

**Objective:**

To evaluate the benefits and risks of the combination of immunotherapy and chemotherapy and to assess the comparative effectiveness of different first-line treatment strategies for extensive-stage SCLC.

**Methods:**

PubMed, Web of Science, EMBASE, and Cochrane Library were searched for randomized clinical trials studying different immunotherapeutics for patients with previously untreated extensive-stage SCLC up to Feb 16, 2020. The primary outcomes were overall survival (OS) and progression-free survival (PFS), and the secondary outcomes were objective response rate (ORR), disease control rate (DCR), and adverse events.

**Results:**

We identified 141 published records, and 4 studies (comprising 2202 patients) were included in the analysis. Immunotherapy (including ipilimumab, atezolizumab, and durvalumab) plus chemotherapy was associated with better OS (hazard ratio (HR) 0.84, 95% confidence interval (CI) 0.75–0.93; risk ratio (RR) 0.90, 95% CI 0.81–1.00) and PFS (HR: 0.81, 95% CI 0.74–0.88; RR 0.96, 95% CI 0.93–0.99) than placebo plus chemotherapy. The addition of immunotherapy to chemotherapy showed similar improvement in ORR, DCR, and adverse events versus placebo plus chemotherapy. On the surface under the cumulative ranking (SUCRA) analysis, the anti-PD-L1 agent, atezolizumab, had the highest likelihood of achieving improved OS (93.4%) and PFS (95.0%).

**Conclusion:**

In the first-line setting, combining immunotherapy with chemotherapy is better than standard chemotherapy in terms of OS and PFS. Across the eligible studies, PD-L1 inhibitors might be preferred. Further explorations of more ICIs in the first-line treatment for extensive-stage SCLC patients should be needed.

## 1. Introduction

Small cell lung cancer represents over 10% of all lung cancer [1]. Extensive-stage SCLC is defined as the cancer cells which extend beyond one hemithorax at the time of initial diagnosis. Platinum-based combination chemotherapy is the current first-line standard-of-care for SCLC. Although the first-line cytotoxic chemotherapy results in an overall response rate with 60%–80%, the majority of extensive-stage SCLC patients suffers disease progression or relapse within months, and the 5-year survival rate is only about 2% [2].

Immunotherapy has revolutionized the treatment strategies for lung cancer. In particular, the cytotoxic T-lymphocyte-associated protein 4 (CTLA-4) and the programmed death-1 (PD-1) signaling pathways have been widely and deeply studied. SCLC has a high rate of gene mutation that indicates SCLC cells may be immunogenic and might respond to immune-related treatments [3–5].

To explore the potential clinical activities of ICI inhibitors in treating patients with extensive-stage SCLC, adding immunotherapy to standard-of-care has been administered as a first-line treatment strategy [6–10]. Two phase III trials indicated that antiprogrammed cell death ligand 1(PD-L1) therapy significantly improved survival outcomes versus platinum-based standard-of-care [9, 10]. Nevertheless, another phase III study of ipilimumab plus chemotherapy failed to show improved efficacy in the first-line treatment of extensive-stage SCLC patients [8]. These results remain controversial and might make it challenging for clinicians to draw any conclusion on which ICI agent is preferred.

Therefore, in this systematic review and network meta-analysis, we aim to evaluate the efficacy and safety of combining immunotherapy with chemotherapy and to compare the benefits and risks of different first-line immunotherapeutic strategies for patients with extensive-stage SCLC.

## 2. Methods

The Preferred Reporting Items for Systematic Reviews and Meta-Analyses (PRISMA) and the PRISMA extension statement for a network analysis were followed, and the details are listed in Table S1 [11, 12].

### 2.1. Search Strategy

PubMed, Web of Science, EMBASE, and Cochrane Library were searched up to Feb 16, 2020, using the following terms: “small-cell lung cancer” OR “small-cell lung cancer” OR “small-cell lung carcinoma” OR “small-cell lung carcinoma” OR “SCLC,” “extensive,” “first-line” OR “first-line,” “nivolumab” OR “pembrolizumab” OR “cemiplimab” OR “atezolizumab” OR “durvalumab” OR “avelumab” OR “ipilimumab” OR “tremelimumab” OR “PD-1 inhibitor” OR “anti-PD-1” OR “anti PD-1” OR “PD-L1 inhibitor” OR “anti-PD-L1” OR “anti PD-L1” OR “CTLA-4 inhibitor” OR “anti-CTLA-4” OR “anti CTLA-4,: and “trial” OR “study” OR “clinical” OR “randomized” OR “randomized” OR “randomly.” No language limitation was performed. Additional clinical studies were checked through reference lists.

### 2.2. Study Selection

Two authors reviewed the records and selected the eligible studies independently. The inclusion criteria were as follows: (1) prospective randomized controlled clinical studies were published in the form of full papers; (2) efficacy and safety data in the studies were extractable; (3) enrolled patients were newly diagnosed as extensive-stage SCLC and previously untreated; and (4) treatment strategies included standard-of-care or monoimmunotherapy or immunotherapy-based combination treatment. Any discrepancies were resolved by discussion. Conference abstracts were not included due to the absence of full data and the potential publication bias. For duplicate studies, the data were available from the most recent and complete publication, and the other reports were used to verify the data.

### 2.3. Data Extraction

Details about the first author's name, publication year, study design, number of patients, registered number, number of patients, mean age, the status of brain metastasis, and interventions were extracted. The primary outcomes were overall survival (OS) and PFS, and the secondary outcomes were ORR, disease control rate (DCR), and adverse events. Hazard ratios (HRs) and associated 95% confidence intervals (CIs) were also extracted for the efficacy evaluation of OS and PFS.

### 2.4. Risk of Bias Assessment

Bi-Cheng Wang and Bo-Ya Xiao independently assessed the risk of bias of the selected studies through the Cochrane Risk of Bias Tool in RevMan 5.3 software (Nordic Cochrane Centre, Copenhagen, Denmark).

### 2.5. Statistical Analysis

All analyses were performed with the frequentist model. STATA statistical software 14.0 (StataCorp, College Station, TX, USA) was used to calculate all outcomes. We conducted network meta-analysis based on a random-effects consistency model to pool evidence from direct and indirect comparisons. Direct and indirect treatment effects were merged into a single effect size, and the relative effects between interventions were presented as risk ratios (RRs) and associated 95% CIs. Different therapeutic strategies were ranked using the surface under the cumulative ranking (SUCRA) probabilities. Higher SUCRA scores indicated greater efficacy. We evaluated the heterogeneity of the results using the chi-squared (*χ*^2^) tests and quantified using *I*^2^ statistic percentages.

## 3. Results

### 3.1. Search Results

**Figure 1:**
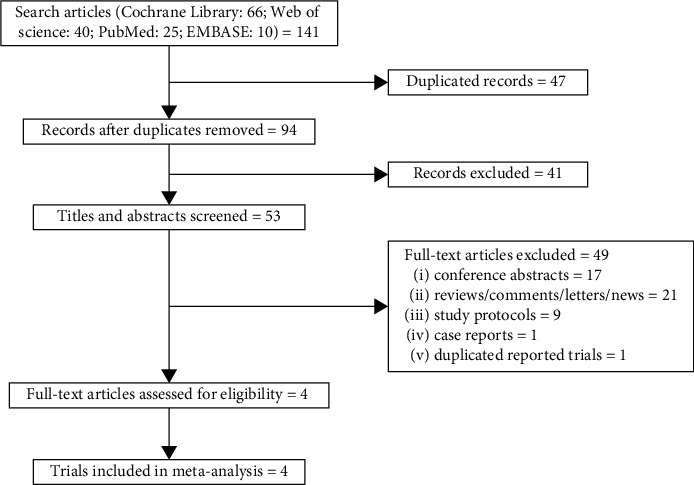
Flow chart of the screening and selection process.

A total of 141 records were included for the initial assessment. 47 duplicates were excluded. After reviewing the titles and abstracts, we further excluded 41 irrelevant records. 53 reports underwent full-text selection. Finally, four articles met the eligibility criteria and were included in the qualitative synthesis and network meta-analysis (Figure 1) [7–10].

### 3.2. Study Characteristics

The basic characteristics of the eligible studies are shown in Table 1. A total of 2202 patients were comprised in the four trials. One study was a phase 2 clinical trial, and the other three were phase 3 trials. Three studies were double-blind clinical trials, and the other one was an open-label trial. All selected studies had been registered on the http://www.clinicaltrials.gov. The mean age of the patients ranged from 57–64 years. Two studies reported ipilimumab, one reported atezolizumab, and the other one reported durvalumab. Chemotherapeutic regimens included etoposide plus platinum (carboplatin or cisplatin) and paclitaxel plus carboplatin.

### 3.3. HRs for OS and PFS

**Figure 2:**
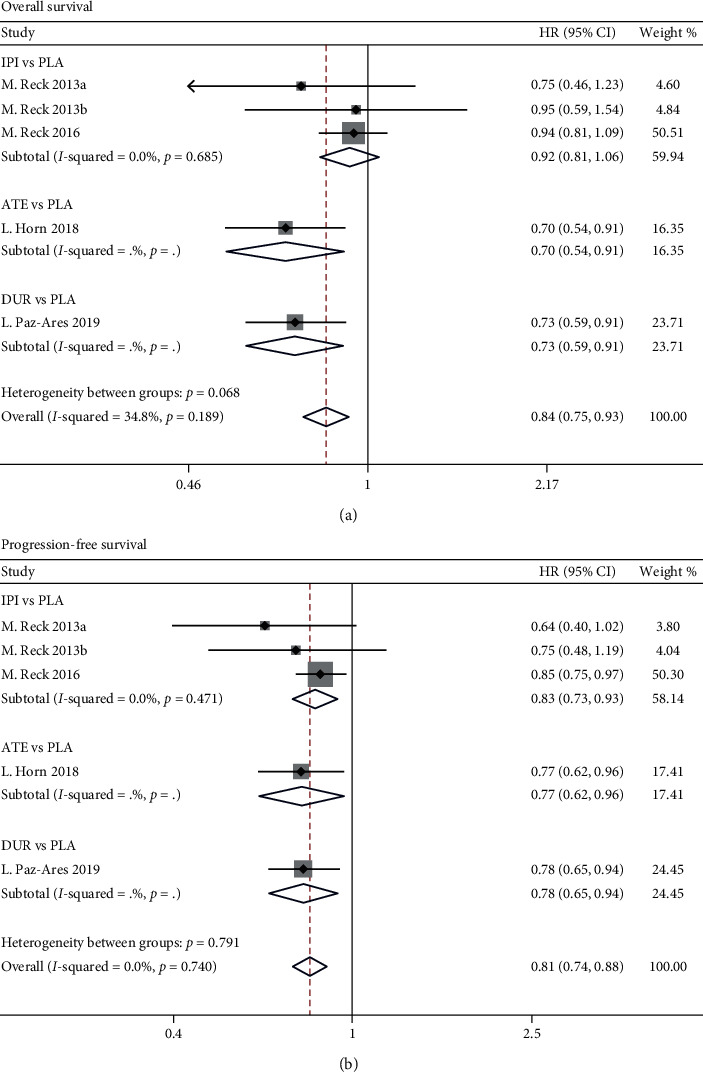
Meta-analyses of the hazard ratios for the included studies examining overall survival (a) and progression-free survival (b) for immunotherapy plus chemotherapy vs. chemotherapy alone. ATE: atezolizumab; DUR: durvalumab; IPI: ipilimumab; PLA: placebo. All these treatments were combined with chemotherapy. (a) indicates phased-IPI, and (b) indicates concurrent-IPI.

The data of HRs were available from all the selected studies. The pooled HRs indicated that immunotherapy plus chemotherapy significantly improved the survival outcomes in terms of OS (HR: 0.84, 95% CI 0.75–0.93, *p*=0.001) and PFS (HR: 0.81, 95% CI 0.74–0.88, *p* < 0.001) (Figure 2).

### 3.4. Network Meta-Analysis of the First-Line Treatment Strategies

**Figure 3:**
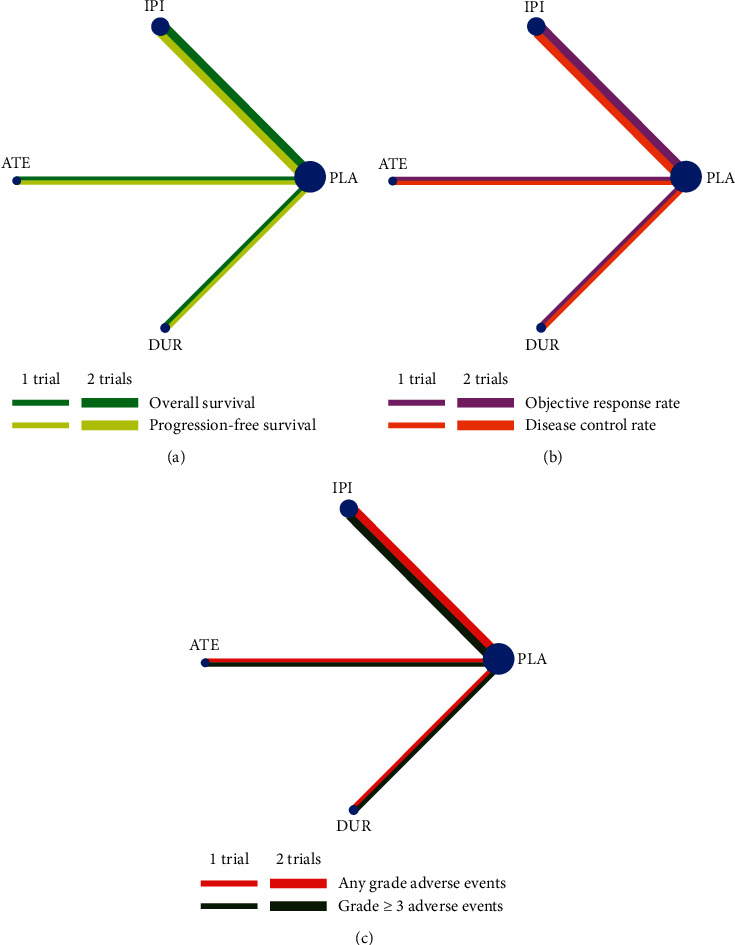
Network meta-analysis of comparisons on different outcomes of first-line treatments in different groups of small cell lung cancer patients. (a) Comparison of overall survival and progression-free survival. (b) Comparison of objective response rate and disease control rate. (c) Comparison of any grade and grade 3 or more adverse events. Direct comparisons are represented by the color lines connecting the treatments. Line width is proportional to the number of trials including every pair of treatments, whereas circle size is proportional to the total number of patients for each treatment in the network. ATE = atezolizumab; DUR = durvalumab; IPI = ipilimumab; PLA = placebo. All these treatments were combined with chemotherapy.

Network meta-analysis included all treatment for OS, PFS, ORR, DCR, and adverse events (Figure 3). In direct comparisons, pooled RRs were 0.90 (95% CI 0.81–1.00) for OS, 0.96 (95% CI 0.93–0.99) for PFS, 1.03 (95% CI 0.98–1.08) for any grade adverse events, and 0.97 (95% CI 0.89–1.05) for grade ≥3 adverse events, showing that the addition of immunotherapy to chemotherapy reduced the risks of death and disease progression and did not increase the risks of adverse events in the first-line treatment of patients with extensive-stage SCLC. However, we found no benefit of combination therapy compared with standard-of-care in terms of ORR (RR: 1.04, 95% CI 0.94–1.16) and DCR (RR: 0.98, 95% CI 0.93–1.02) (Table 2).

**Figure 4:**
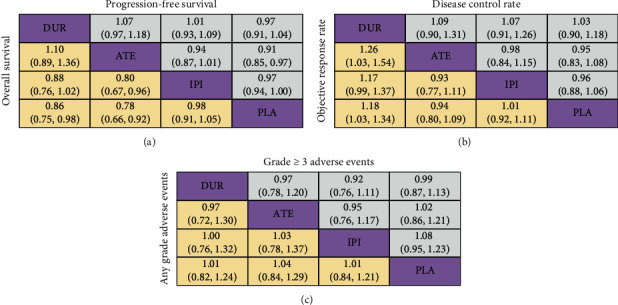
Risk ratios for the pairwise comparisons of the network meta-analysis. Direct and indirect comparisons should be read from left to right. For overall survival, progression-free survival, and adverse events, a risk ratio of less than 1 favors the left treatment. For the objective response rate and disease control rate, a risk ratio of less than 1 favors the right treatment. ATE indicates atezolizumab; DUR indicates durvalumab; IPI indicates ipilimumab; PLA indicates placebo. All these treatments were combined with chemotherapy.

**Figure 5:**
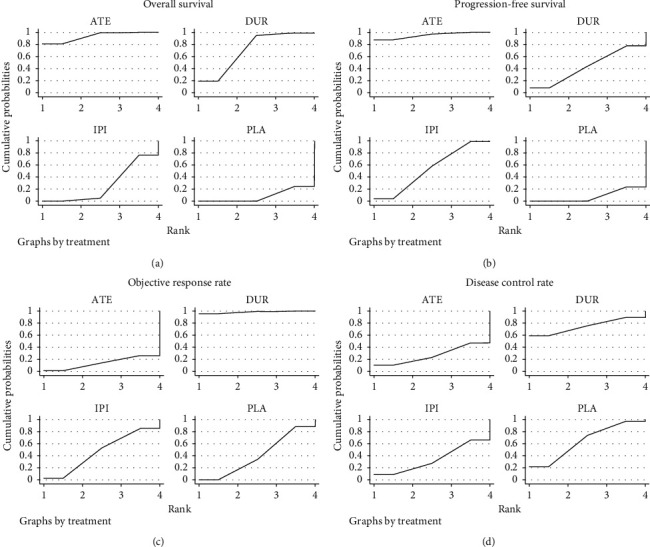
Ranking probabilities of the different comparisons for overall survival (a), progression-free survival (b), objective response rate (c), and disease control rate (d). ATE means atezolizumab; DUR means durvalumab; IPI means ipilimumab; PLA means placebo. All these treatments were added to chemotherapy.

In the pairwise comparisons, atezolizumab had a superior effect on improving the risk of death compared with ipilimumab (RR 0.80, 95% CI 0.67–0.96). However, in terms of disease progression, response rate, and toxicities, slight differences were showed across the first-line treatment strategies (Figure 4). Furthermore, on SUCRA analysis, we found that atezolizumab was ranked highest in terms of OS (93.4%) and PFS (95.0%), while durvalumab was ranked highest in terms of ORR (98.3%) and DCR (74.8%) (Figure 5 and Table 3).

### 3.5. Risk of Bias Assessment

**Figure 6:**
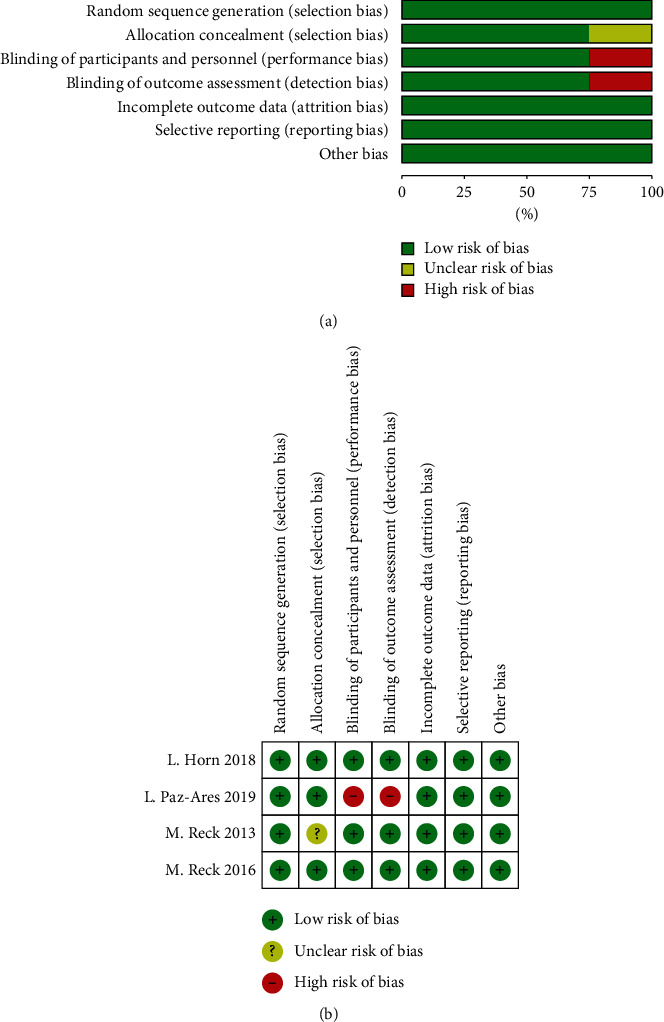
Risk of bias graph (a) and summary (b).

All the selected studies had achieved randomization. The allocation concealment was unclear in only one trial. Overall, the trials were deemed to be at low-to-moderate risk for bias, for which three trials had blinding of participant and personnel and blinding of outcome assessment, whereas the other one was not blinded (Figure 6).

## 4. Discussion

In this study, we found that immunotherapy combined with standard-of-care significantly improves survival outcomes for previously untreated patients with extensive-stage SCLC against standard-of-care only. Moreover, anti-PD-L1 therapy might be superior to anti-CTLA-4 therapy in the first-line treatment for extensive-stage SCLC.

CTLA-4, cytotoxic T-lymphocyte antigen-4, is a negative regulator of T-cell activation [13]. Ipilimumab and tremelimumab are anti-CTLA-4 monoclonal antibodies. Blocking the interaction of CTLA-4 with its ligands (CD80/CD86) could overcome the blockage of T-cell activation and proliferation [14–16]. Clinical trials with CTLA-4 inhibitors have shown durable tumor responses in multiple cancer types [17–22]. In 2013, M. Reck found that ipilimumab plus paclitaxel and carboplatin appeared to prolong OS and PFS in previously untreated extensive-stage SCLC patients [7]. However, in the phase 3 randomized trial reported by M. Reck in 2016, the addition of ipilimumab to etoposide and platinum failed to result in a statistically significant improvement in OS, with a median OS of 11.0 months versus 10.9 months in the chemotherapy group [8]. Therefore, during recent years, researchers and clinicians have paid attention to augment the efficacy of anti-CTLA-4 therapy.

There are several studies in detecting the benefits and risks of the combination of CTLA-4 and PD-1 inhibitors in SCLC [23–25]. A multicenter, open-label, phase 1/2 trial CheckMate-032 showed that the ORR was 21.9% when recurrent SCLC patients received ipilimumab and nivolumab combination therapy. However, this study did not discover the improvement of median OS by the combination therapy [23]. The ADRIATIC study, an ongoing randomized controlled phase 3 trial, compares tremelimumab plus durvalumab with durvalumab in treating limited-stage SCLC patients who have finished concurrent chemoradiotherapy [24]. We are eagerly waiting for the results of this study. Perhaps in the future study, chemotherapy combined with anti-CTLA-4 and anti-PD-1 therapies might be one of the therapeutic modalities for chemotherapy-naive SCLC patients.

Clinical studies on programmed cell death-1 (PD-1) and PD-L1 inhibitors in curing SCLC are still limited. For relapsed or refractory SCLC patients treated with pembrolizumab combined with paclitaxel, the ORR was 23.1%, with a median OS of 9.1 months and a median PFS of 5.0 months [26]. In a phase 1 trial, the median OS and PFS were 8.4 months and 6.1 months, respectively, when extensive-stage SCLC patients were administered induction chemotherapy followed by pembrolizumab and radiotherapy [27]. According to the results of CheckMate32, nivolumab monotherapy achieved a median OS of 5.7 months in recurrent SCLC [23]. In the first-line treatment of extensive-stage SCLC, PD-L1 inhibitors exhibited slightly better results. Atezolizumab plus chemotherapy achieved a median OS of 12.3 months and a median PFS of 5.2 months versus 10.3 months and 4.3 months in the chemotherapy group [9]. However, durvalumab plus platinum-etoposide was also associated with an improvement in OS (13.0 months versus 10.3 months) but not PFS (5.1 months versus 5.4 months) as the first-line treatment strategy [10]. Another phase 3 trial KEYNOTE-604 has not been published yet [6]. We are expecting this study to bring us new findings.

Additionally, several clinical trials have studied the efficacy and safety of immune checkpoint inhibitors (ICIs) in the second-line therapy for SCLC; however, a little progress has been made. In the phase 1b study KEYNOTE-028 and the phase 2 study KEYNOTE-158, SCLC patients who had been administered two or more lines of previously systemic therapeutics received pembrolizumab, the objective response rate (ORR) was 19.3% and the incidence of grade ≥3 adverse events was 9.6% [28, 29]. A phase 1 study assessed the safety of combining pembrolizumab with radiotherapy for extensive-stage SCLC patients who had completed chemotherapy and showed that the combination treatment was tolerated well, with a median progression-free survival (PFS) of 6.1 months [27]. Another phase 2 trial to explore the efficacy of pembrolizumab plus chemotherapy for extensive-stage SCLC is ongoing (NCT02359019). In the phase 1/2 study CheckMate-032, advanced SCLC patients achieved an ORR of 11.9% after the third- or later-line nivolumab monotherapy treatment [25, 30, 31].

Although the results of ICIs in the first-line treatment of extensive-stage SCLC vary from each other, the addition of immunotherapy to chemotherapy did improve the survival outcomes. However, in our analysis, the combination therapy did not significantly increase the ORR (RR 1.04, 95% CI 0.94–1.16, *p*=0.425) and DCR (RR 0.98, 95% CI 0.93–1.02, *p*=0.316) but reduced the risk of death and disease progression compared with chemotherapy alone. In the SUCRA analysis, durvalumab showed the highest ORR (98.3%) and DCR (74.8%). Nevertheless, based on the pairwise comparisons, durvalumab might only be better than ipilimumab in terms of OS (RR 0.88, 95% CI 0.76–1.02) but not PFS (RR 1.01, 95% CI 0.87–1.01). When comparing each combination therapy with chemotherapy alone, we found no significant differences. Moreover, the addition of durvalumab, atezolizumab, or ipilimumab to chemotherapy did not increase adverse events. We convince that platinum-based chemotherapy is greatly important for extensive-stage SCLC patients to achieve a high response rate. Although adding immunotherapy to chemotherapy failed to improve the responses, combination therapy prolonged the survival time without increasing adverse events, and we suppose that the administration of ICIs might increase the sensitivity of tumor cells to cytotoxic chemotherapy [32].

## 5. Limitation

Three standard-of-care strategies, etoposide plus cisplatin, etoposide plus carboplatin, and paclitaxel plus carboplatin, were comprised in the analysis. Up to now, no solid evidence could certify the impact of different chemotherapeutic regimens on the effects of immunotherapy. Additionally, there were only four studies including 2202 SCLC patients and three ICIs. Thus, more data on clinical trials and other inhibitors are needed to complement our results.

## 6. Conclusion

Immunotherapy combined with standard-of-care could be a first-line treatment option for patients with extensive-stage SCLC, without increasing toxicities. Further explorations are warranted to detect the efficacy and safety of anti-PD-L1 therapy and whether PD-1 inhibitors are noninferior or superior to PD-L1 inhibitors.

## Figures and Tables

**Table 1:** Basic characteristics of the clinical trials in the analysis.

Study	Publication year	Design	Registered number	No. of patients	No. of males	Mean age (range, year)	Brain metastasis	Immunotherapy	Chemotherapy
M. Reck	2013	A randomized, double-blind, multicenter phase 2 trial	NCT00527735	130	98	57–59	NR	Ipilimumab	Paclitaxel and carboplatin

M. Reck	2016	A randomized, double-blind, multicenter phase 3 trial	NCT01450761	1132	758	62–63	NR	Ipilimumab	Etoposide and platinum

L. Horn	2018	A randomized, double-blind, multicenter, phase 1 (safety) and phase 3 (efficacy) trial	NCT02763579	403	261	64	35	Atezolizumab	Etoposide and carboplatin

L. Paz-Ares	2019	A randomized, open-label, multicenter phase 3 trial	NCT03043872	537	374	62–63	55	Durvalumab	Etoposide and platinum

NR not reported.

**Table 2:** Pooled risk ratios of the selected trials.

Study	RR	95% CI	*p* value
Overall survival	0.90	0.81–1.00	0.054
Progression-free survival	0.96	0.93–0.99	0.002
Objective response rate	1.04	0.94–1.16	0.452
Disease control rate	0.98	0.93–1.02	0.316
Any grade adverse events	1.03	0.98–1.08	0.202
Grade 3 ≥ adverse events	0.97	0.89–1.05	0.427

RR indicates risk ratio; 95% CI indicates 95% confidence interval.

**Table 3:** Ranking probabilities of different first-line treatment strategies.

Strategy	SUCRA% for OS	SUCRA% for PFS	SUCRA% for ORR	SUCRA% for DCR
ATE	93.4	95.0	13.9	26.9
DUR	71.1	43.4	98.3	74.8
IPI	27.2	53.6	47.0	34.1
PLA	8.4	8.1	40.8	64.2

SUCRA = surface under the cumulative ranking; OS = overall survival; PFS = progression-free survival; ORR = objective response rate; DCR = disease control rate; ATE = atezolizumab; DUR = durvalumab; IPI = ipilimumab; PLA = placebo. All these treatments were combined with chemotherapy.

## Data Availability

The data used to support the findings of this study are available from the corresponding author upon request.

## Additional Points

*Highlights*. (1) Immunotherapy (including ipilimumab, atezolizumab, and durvalumab) plus chemotherapy was associated with better OS and PFS than chemotherapy alone for patients with extensive-stage SCLC. (2) Atezolizumab had the highest likelihood of achieving improved OS and PFS. (3) The addition of immunotherapy to chemotherapy showed similar improvement in ORR, DCR, and adverse events versus placebo plus chemotherapy.

## Supplementary Materials

Supplementary Materials Table S1: checklist of items to include when reporting a systematic review involving a network meta-analysis.
