# Corrigendum to “Efficacy and Safety of First-Line Immunotherapy in Combination with Chemotherapy for Patients with Extensive-Stage Small Cell Lung Cancer: A Systematic Review and Network Meta-Analysis”

**DOI:** 10.1155/2021/3735726

**Published:** 2021-02-25

**Authors:** Bi-Cheng Wang, Bo-Ya Xiao, Peng-Cheng Li, Bo-Hua Kuang, Wang-Bing Chen, Pin-Dong Li, Guo-He Lin, Quentin Liu

**Affiliations:** ^1^Cancer Center, Union Hospital, Tongji Medical College, Huazhong University of Science and Technology, Wuhan 430022, China; ^2^Shanghai Eastern Hepatobiliary Surgery Hospital, Shanghai 200438, China; ^3^Department of Oncology, The Second Affiliated Hospital of Anhui Medical University, Hefei 230601, China; ^4^State Key Laboratory of Oncology in South China, Collaborative Innovation Center for Cancer Medicine, Cancer Center, Sun Yat-sen University, Guangzhou 510060, China

In the article titled “Efficacy and Safety of First-Line Immunotherapy in Combination with Chemotherapy for Patients with Extensive-Stage Small Cell Lung Cancer: A Systematic Review and Network Meta-Analysis” [1], the information was omitted in the Acknowledgments section: the Hubei Provincial Natural Science Foundation (grant number: 2020 to Bi-Cheng Wang). The corrected section appears as follows:
